# Rhodomyrtone-rich fractions from *Rhodomyrtus tomentosa* (Aiton) Hassk. leaves: A potent antimicrobial compound for *Staphylococcus pseudintermedius*

**DOI:** 10.14202/vetworld.2025.1025-1035

**Published:** 2025-04-30

**Authors:** Mareena Daus, Supakit Paosen, Sakkarin Lethongkam, Suda Chakthong, Supayang Piyawan Voravuthikunchai

**Affiliations:** 1 Center of Antimicrobial Biomaterial Innovation-Southeast Asia, Faculty of Science, Prince of Songkla University, Hat Yai, Songkhla, Thailand; 2 Division of Biological Science, Faculty of Science, Prince of Songkla University, Hat Yai, Songkhla, Thailand; 3 Division of Physical Science, Faculty of Science, Prince of Songkla University, Hat Yai, Songkhla, Thailand

**Keywords:** antibacterial activity, biofilm, herbal medicine, rhodomyrtone, *Rhodomyrtus tomentosa*, *Staphylococcus pseudintermedius*

## Abstract

**Background and Aim:**

*Staphylococcus pseudintermedius* is an opportunistic zoonotic pathogen frequently implicated in skin and wound infections in companion animals. Its ability to form biofilms complicates treatment by increasing antibiotic resistance. Rhodomyrtone, a potent acylphloroglucinol isolated from *Rhodomyrtus tomentosa*, exhibits promising antibacterial activity against Gram-positive bacteria. This study aimed to develop rhodomyrtone-rich fractions and evaluate their antibacterial and antibiofilm activities against *S. pseudintermedius*.

**Materials and Methods:**

Ethanolic extracts of *R. tomentosa* leaves were subjected to acetone partitioning followed by quick column chromatography, yielding fractions F1–F15. Fractions F3–F7 were selected based on thin-layer chromatography and ^1^H nuclear magnetic resonance for rhodomyrtone content and quantified by high-performance liquid chromatography. Antibacterial activity against *Staphylococcus aureus* American Type Culture Collection (ATCC) 25923, *S. pseudintermedius* ATCC 49444, and 10 clinical *S. pseudintermedius* isolates was assessed using broth microdilution to determine minimal inhibitory concentration (MIC) and minimal bactericidal concentration (MBC) values. Fraction F4, with the highest rhodomyrtone content, was further investigated using time-kill kinetics, scanning electron microscopy (SEM), and a crystal violet assay for biofilm inhibition.

**Results:**

Fraction F4 contained the highest rhodomyrtone concentration (489.08 mg/g) and demonstrated the most potent antibacterial activity, with MIC and MBC values ranging from 0.5 to 2 µg/mL and 2 to 8 µg/mL, respectively, against clinical isolates. The time-kill study revealed a 4-log reduction (99.99%) in bacterial load within 8 h at 2× MIC. Biofilm formation by all tested *S. pseudintermedius* isolates was significantly inhibited at sub-MIC concentrations of F4 (p < 0.05). SEM analysis showed notable morphological disruptions in bacterial cells treated with F4, suggesting membrane damage as a possible mechanism of action.

**Conclusion:**

Fraction F4 from *R. tomentosa* leaf extract exhibited strong antibacterial and antibiofilm activity against *S. pseudintermedius*, comparable to that of pure rhodomyrtone and superior to doxycycline. These findings support the potential use of rhodomyrtone-rich fractions as standardized herbal antibacterial agents in veterinary medicine, providing an effective alternative for treating drug-resistant staphylococcal infections.

## INTRODUCTION

*Staphylococcus pseudintermedius*, first identified as a coagulase-positive staphylococcal species in 2005, commonly colonizes the skin and mucous membranes of companion animals, particularly dogs and cats, and is frequently associated with wound infections [1, 2]. This opportunistic pathogen has been isolated from multiple anatomical sites, including the nares, mouth, pharynx, forehead, groin, and anus of healthy animals. Moreover, human infections have been reported following direct contact with infected animals, indicating zoonotic transmission [3-5]. Biofilm formation is widely recognized as a key virulence factor that complicates treatment for several coagulase-positive staphylococci, including *S. pseudintermedius* [6, 7]. On adherence to tissue surfaces, bacteria form biofilms comprising communities encased in extracellular polysaccharide matrices. These structures enhance bacterial survival by impeding both host immune responses and the efficacy of antimicrobial agents [8]. Infections associated with biofilms are particularly difficult to treat, as biofilm-embedded bacteria exhibit increased resistance to antibiotics and a heightened ability to evade immune clearance compared with their planktonic counterparts [9].

*Rhodomyrtus tomentosa* (Aiton) Hassk., a flowering species of the *Myrtaceae* family native to Southern and Southeast Asia, has long been utilized in traditional medicine to treat ailments such as urinary tract infections, diarrhea, and dysentery, and to support immune function [10, 11]. Extracts derived from its leaves have demonstrated antioxidant, antimicrobial, and anticancer activities [12-14]. The plant is a natural source of rhodomyrtone, an acylphloroglucinol compound with potent antibacterial effects against a broad spectrum of Gram-positive bacteria [15, 16]. Rhodomyrtone also exhibits anti-inflammatory, anticancer, and antidepressant properties [17-20].

Despite extensive evidence supporting the antimicrobial efficacy of *R. tomento*sa leaf extracts and its bioactive compound rhodomyrtone against various Gram-positive bacteria, research has primarily focused on crude extracts or purified compounds. Most studies have not investigated the intermediate rhodomyrtone-rich fractions, which could offer a more practical and cost-effective approach to formulation and standardization of herbal therapeutics. Furthermore, while *S. pseudintermedius* is increasingly recognized as a multidrug-resistant zoonotic pathogen with significant clinical relevance in veterinary medicine, there is a notable lack of studies evaluating the antibacterial and antibiofilm properties of *R. tomento*sa derivatives specifically against this organism. No previous reports have comprehensively assessed the efficacy of rhodomyrtone-rich fractions in inhibiting both planktonic growth and biofilm formation of *S. pseudintermediu*s, which represents a critical barrier in the development of effective alternative therapies.

The present study aims to address this gap by developing and characterizing rhodomyrtone-rich fractions from the ethanolic extract of *R. tomento*sa leaves using chromatographic and spectroscopic techniques. These fractions were systematically evaluated for their antibacterial activity against *S. pseudintermediu*s clinical isolates and standard strains, with particular emphasis on determining minimum inhibitory and bactericidal concentrations. The most active fraction was further assessed for its bactericidal kinetics, morphological effects using scanning electron microscopy (SEM), and its ability to inhibit biofilm formation. This research seeks to explore the potential of rhodomyrtone-rich fractions as standardized, plant-based alternatives to conventional antibiotics for managing infections caused by *S. pseudintermediu*s.

## MATERIALS AND METHODS

### Ethical approval

This study received approval from the Institutional Biosafety Committee of Prince of Songkla University (Approval No. IBC.PSU.002-2025).

### Study period and location

The research was conducted from May 2024 to January 2025. The maceration and isolation of *R. tomentosa* leaves were carried out at Chemistry Building, Faculty of Science, Prince of Songkla University. Antibacterial activity assessments were performed at Center of Antimicrobial Biomaterial Innovation-Southeast Asia, Faculty of Science, Prince of Songkla University.

### Chemicals

Mueller–Hinton broth and Mueller–Hinton agar, along with other culture media and supplements, were procured from Gibco (Thermo Fisher Scientific, USA). Additional reagents and chemicals were obtained from Sigma-Aldrich (USA) and Merck (USA). All chromatographic and extraction solvents (P.S. Science Chemical Ltd., Part., Thailand) were distilled before use at their respective boiling points.

### Microorganisms and culture conditions

Reference strains used in this study included *Staphylococcus aureus* American Type Culture Collection (ATCC) 25923 and *S. pseudintermedius* ATCC 49444 (ATCC, USA). Ten clinical isolates of *S. pseudintermedius* (CABI 240101-204110) were collected from skin and wound infections in animals presented at the Veterinary Teaching Hospital, Prince of Songkla University, Thailand. Identification was performed using standard biochemical assays, including coagulase and urease activity, acetoin production, polymyxin B resistance, and acid production from mannitol and glucose. Antibiotic susceptibility testing was conducted using the Kirby–Bauer disk diffusion method following Clinical and Laboratory Standards Institute (CLSI) guidelines [21], with antibiotics including chloramphenicol, clindamycin, tetracycline, clarithromycin, ciprofloxacin, and trimethoprim-sulfamethoxazole. All isolates were subcultured on Mueller–Hinton agar at 37°C overnight.

### Extraction and isolation

*R. tomentosa* leaves were collected from Phatthalung Province, Thailand, in December 2023. A voucher specimen (NPRC0057) was deposited at the Faculty of Traditional Thai Medicine, Prince of Songkla University [16]. The extraction procedure was adapted from a previously published method [22]. Briefly, 800× *g* of ground, dried leaves were macerated in 95% ethanol at room temperature (25°C) for 7 days, repeated 3 times. The pooled filtrates were concentrated using a rotary evaporator (BUCHI Rotavapor R-200, Switzerland). The resulting ethanolic extract was partitioned with acetone to yield acetone-soluble and acetone-insoluble fractions. The acetone-soluble fraction was further subjected to quick column chromatography on silica gel 60 (Merck) using isocratic elution with hexane: ethyl acetate (92:8 v/v), yielding 15 fractions (F1–F15). Thin-layer chromatography (TLC) analysis (Merck) identified rhodomyrtone in fractions F3-F7. The presence of rhodomyrtone was further confirmed by comparing TLC profiles with pure rhodomyrtone and via ^1^H nuclear magnetic resonance (^1^H NMR, Bruker, Germany) analysis [23].

### Quantitative analysis of rhodomyrtone in fractions F3-F7

Fractions F3–F7 were dissolved in 0.5 mL of dimethyl sulfoxide (DMSO) and diluted 20-fold with acetonitrile/0.1% phosphoric acid in water (7:3 v/v). Rhodomyrtone content was quantified using high-performance liquid chromatography (HPLC) on an Agilent 1100 system equipped with a variable-wavelength detector (Agilent, USA). Separation was carried out on a Zorbax Eclipse XDB C-8 reverse-phase column (Agilent, USA) under isocratic elution (ACN/0.1% phosphoric acid in water, 7:3 v/v) at a flow rate of 1.2 mL/min. The column temperature was maintained at 35°C.

### Minimal inhibitory concentration (MIC) and minimal bactericidal concentration (MBC)

The antibacterial activity of *R. tomentosa* extract and rhodomyrtone-rich fractions (F3–F7) was evaluated against Gram-positive pathogens including *S. aureus* and *S. pseudintermedius*, following CLSI guidelines [21]. The broth microdilution method was conducted in 96-well microtiter plates. Bacterial suspensions were adjusted to 10^6^ colony forming unit (CFU)/mL, and 100 µL of each suspension was added to wells containing test compounds at final concentrations ranging from 0.125 to 1,024 µg/mL. Plates were incubated at 37°C for 16–18 h. Doxycycline, commonly used to treat wound infections in companion animals, was included as a reference control [24, 25]. MIC was defined as the lowest concentration that completely inhibited visible bacterial growth. MBC was determined using the drop plate method and defined as the lowest concentration at which no colony formation was observed on the culture medium after 24 h of incubation.

### Time-kill kinetics assay

*S. pseudintermedius* CABI 240105 was selected as the representative strain for time-kill kinetics analysis. The assay was conducted based on previously published protocols with minor modifications [26]. Bacterial suspensions at 10^6^ CFU/mL were treated with fraction F4 at concentrations of 1×, 2×, and 4× MIC (MIC = 0.5 µg/mL) and incubated at 37°C. Samples were collected at 0, 2, 4, 6, 8, 12, 18, and 24 h, serially diluted ten-fold, and plated on tryptic soy agar using the drop plate method. Plates were incubated at 37°C for 24 h. The negative control consisted of 1% DMSO. All experiments were performed in triplicate.

### SEM

SEM was employed to observe the morphological effects of fraction F4 on *S. pseudintermedius* CABI 240105 [27]. Bacterial suspensions (10^6^ CFU/mL) were incubated for 24 h on 1 × 1 cm sterile glass slides in 24-well plates. Cells were treated with fraction F4 at 2× MIC (0.5 µg/mL) for 3 h. After treatment, samples were washed twice with phosphate-buffered saline (PBS) and fixed in 2.5% glutaraldehyde for 24 h. Slides were dehydrated through a graded ethanol series, dried, coated with gold, and visualized using a scanning electron microscope to assess morphological changes.

### Biofilm formation

Biofilm formation by *S. pseudintermedius* clinical isolates was assessed using polystyrene microtiter plates, following the protocol by Saising *et al*. [15]. Bacterial cultures were grown in tryptic soy broth supplemented with 0.25% glucose and incubated at 37°C for 24 h. *S. aureus* ATCC 25923 served as a positive control, while 1% DMSO was used as the negative control. After incubation, wells were washed twice with PBS to remove non-adherent cells, air-dried, and stained with 200 µL of 0.1% crystal violet for 30 min. The excess stain was removed by washing with PBS. The stained biofilms were solubilized in DMSO, and absorbance was measured at 570 nm using a microplate reader. The percentage of biofilm formation was calculated as ([optical density (OD) of treated well/OD of control well] × 100).

### Statistical analysis

All experiments were independently performed in triplicate. Data are expressed as mean ± standard deviation. Statistical analysis was conducted using one-way analysis of variance, followed by Dunnett’s test. A p < 0.05 was considered statistically significant.

## RESULTS

### Extraction and isolation

The ethanolic extraction of ground-dried *R. tomentosa* leaves yielded 40.96 g of a dark green gum, corresponding to a yield of 5.12%. The extract was further fractionated to obtain five rhodomyrtone-rich fractions (F3–F7), as determined by TLC and ^1^H NMR spectroscopy.

**Figure 1:**
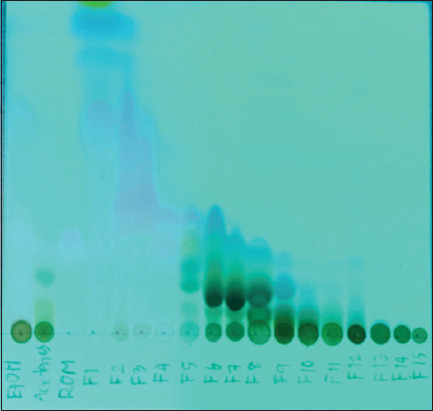
Thin-layer chromatography (TLC) characterization of ethanol extract (EtOH), acetone soluble (Acetone S), standard rhodomyrtone, and fractions F1-F15 (F1-F15). The plate was placed in the TLC chamber (contained mobile phase: Hexane: EtOAc; 92:8) and covered by a lid, and it was left until the mobile phase reached the upper line. Spots were visualized with a positive result in the ultraviolet detection box at 254 nm.

TLC analysis of the ethanolic extract, the acetone-soluble fraction, and fractions F3–F7 revealed chromatograms identical to that of pure rhodomyrtone, with an Rf value of 0.34 (Figure 1).

**Figure 2:**
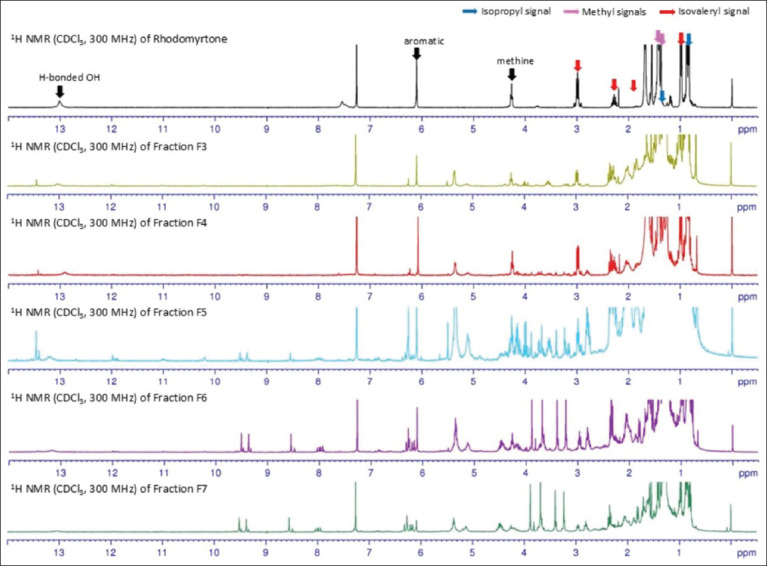
^1^H nuclear magnetic resonance (NMR) spectra of rhodomyrtone and fractions F3–F7. All spectra were recorded using CDCl_3_ and analyzed using a NMR Bruker instrument (300 MHz).

The ^1^H NMR spectra of fractions F3–F5 (Figure 2) confirmed the presence of rhodomyrtone, showing characteristic signals for hydrogen-bonded hydroxy protons, aromatic protons, methine protons, and methyl groups, as well as isovaleryl and isopentyl side chains, consistent with pure rhodomyrtone [23]. Although fractions F6 and F7 also exhibited TLC profiles similar to rhodomyrtone, their ^1^H NMR spectra indicated the presence of multiple other compounds, suggesting only minor quantities of rhodomyrtone in these fractions.

### Quantitative analysis of fractions F3–F7

**Figure 3:**
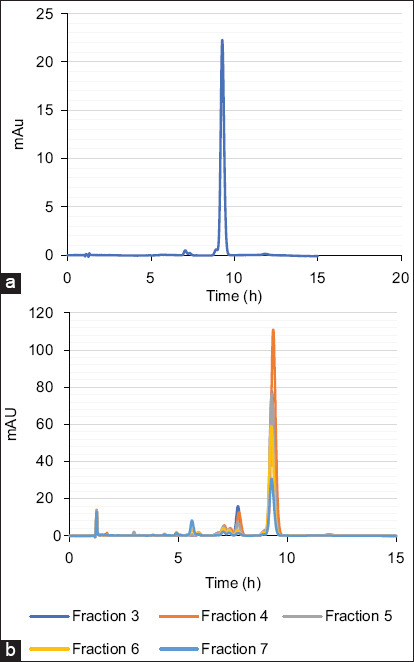
High-performance liquid chromatography chromatograms of (a) standard rhodomyrtone (b) fractions F3–F7 compared with the standard rhodomyrtone. The details of the chromatographic analysis can be found in the experiment.

The rhodomyrtone content in fractions F3–F7 was quantified using HPLC. Figure 3 displays chromatograms of the fractions compared with the rhodomyrtone standard. The method demonstrated high reliability, with a calibration curve showing a coefficient of determination (R^2^) of 0.9999. Rhodomyrtone was detected at a retention time of 9.3 min in all fractions. Fraction F4 exhibited the highest rhodomyrtone concentration at 489.08 mg/g, approximately 50% w/w. The rhodomyrtone content in fractions F3, F5, F6, and F7 was 217.12, 242.56, 233.83, and 119.63 mg/g, respectively.

### Antibacterial activity of fractions

The antibacterial activity of the ethanolic extract and its fractions was assessed using the broth microdilution method in accordance with CLSI guidelines, targeting *S. aureus* ATCC 25923, *S. pseudintermedius* ATCC 49444, and a clinical isolate, *S. pseudintermedius* CABI 240105. The MIC and MBC of doxycycline were 0.125 and 0.25 µg/mL for *S. aureus* ATCC 25923, 0.06, and 0.25 µg/mL for *S. pseudintermedius* ATCC 49444, and 4 and 8 µg/mL for the clinical isolate CABI 240105, respectively. The ethanolic extract exhibited MIC and MBC values of 16 and 256 µg/mL against *S. aureus*. All fractions demonstrated greater antibacterial potency than the crude extract, with MICs and MBCs ranging from 0.5 to 8 µg/mL.

Against *S. pseudintermedius* ATCC 49444, the ethanolic extract and acetone-soluble fraction showed MICs of 8 µg/mL and MBCs of 128 µg/mL and 64 µg/mL, respectively. Fractions F3–F7 demonstrated enhanced activity, with MICs and MBCs ranging from 0.25 to 1 µg/mL and 2 to 8 µg/mL, respectively. For the clinical isolate CABI 240105, the extract yielded MIC and MBC values of 16 and 64 µg/mL, while fractions F4 and F5 exhibited the strongest antibacterial effects (MIC: 0.5 µg/mL, MBC: 2 µg/mL). Fractions F3 and F6 had MICs and MBCs of 1 and 4 µg/mL, respectively. Among all fractions, F7 was the least active, with MIC and MBC values of 2 and 16 µg/mL. Nevertheless, the antibacterial activity of fractions F3–F7 was comparable to that of doxycycline. The findings are summarized in Table 1.

**Table 1:** MIC and MBC of the *Rhodomyrtus tomentosa* extracts against staphylococci.

Antibacterial agents	MIC/MBC (µg/mL)		

	*Staphylococcus pseudintermedius* CABI 240105	*Staphylococcus pseudintermedius* ATCC 49444	*Staphylococcus aureus* ATCC 25923
EtOH extract	16/64	8/128	16/256
Acetone solution	16/64	8/64	16/1024
Fraction F3	1/4	1/8	1/2
Fraction F4	0.5/2	0.5/2	0.5/1
Fraction F5	0.5/2	0.25/2	0.5/2
Fraction F6	1/4	0.25/2	0.5/2
Fraction F7	2/16	1/2	2/8
Doxycycline	4/8	0.06/0.25	0.125/0.25

MIC=Minimum inhibitory concentration, MBC=Minimum bactericidal concentration, ATCC=American Type Culture Collection

### Aaa

**Antibacterial activity against clinical isolates of *S. pseudintermedius***

Given its high rhodomyrtone content and potent antibacterial activity, fraction F4 was selected for further investigation. Its antibacterial efficacy was evaluated against ten clinical isolates of *S. pseudintermedius* (Table 2). The MICs and MBCs ranged from 0.5 to 2 µg/mL and 2 to 8 µg/mL, respectively. Fraction F4 inhibited all clinical isolates effectively, with MIC_50_ and MIC_90_ values of 0.5 µg/mL and MBC_50_ and MBC_90_ values of 4 µg/mL. These results were consistent with those of the doxycycline control, which had MIC and MBC ranges of 0.5–4 µg/mL and 1–8 µg/mL, respectively.

**Table 2:** MIC and MBC of the fraction F4 against clinical isolates of *Staphylococcus pseudintermedius*.

Strains or isolates	Fraction F4						Doxycycline					
	
	MIC (µg/mL)			MBC (µg/mL)			MIC (µg/mL)			MBC (µg/mL)		
			
	Range	MIC_50_	MIC_90_	Range	MBC_50_	MBC_90_	Range	MIC_50_	MIC_90_	Range	MBC_50_	MBC_90_
*Staphylococcus pseudintermedius* (n = 10)	0.5–2	0.5	0.5	2–8	4	4	0.5–4	0.5	4π	1–8	1	8
*Staphylococcus pseudintermedius* ATCC 49444	1			8			0.125			0.25		
*Staphylococcus aureus* ATCC 25923	0.5			1			0.125			0.25		

MIC=Minimum inhibitory concentration, MBC=Minimum bactericidal concentration, MIC_50_ and MIC_90_ values are defined as the lowest concentration of antibacterial agents at which 50% and 90% of the isolates were inhibited, respectively. MBC_50_ and MBC_90_ values were defined as the lowest concentration of antibaterial agents at which 50% and 90% of the isolated were killed, respectively

### Time-kill kinetics

**Figure 4:**
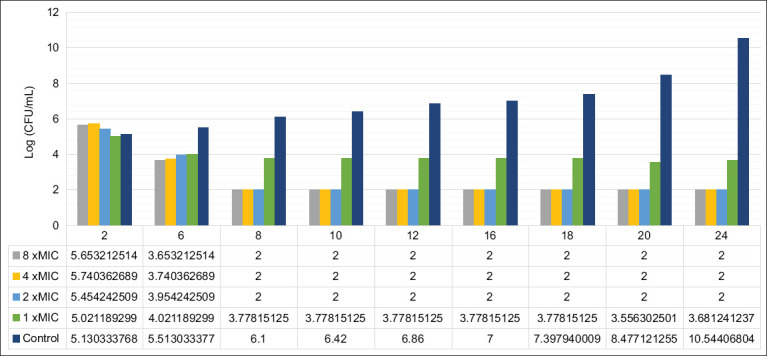
Time-kill kinetics of F4 fraction against representative *Staphylococcus pseudintermedius* isolates. The bacteria were treated with the fraction F4 at different concentration at 8× minimal inhibitory concentration (MIC), 4× MIC, 2× MIC, and 1× MIC. The fraction F4 against *S. pseudintermedius* CABI 240105 with an MIC of 0.5 µg/mL. 1% dimethylsulfoxide was used as a negative control. Each symbol indicates the mean ± standard deviation.

A time-kill kinetics assay was performed to determine the relationship between concentration and bactericidal effect. While the activity of rhodomyrtone against *S. aureus* has previously been described [15], its effect on *S. pseudintermedius* had not been reported. The clinical isolate CABI 240105 was treated with various concentrations of fraction F4, and bacterial counts were recorded at 2-h intervals over 24 h (Figure 4). After 6 h, significant growth reduction was observed at all concentrations tested (1×, 2×, 4×, and 8× MIC, MIC = 0.5 µg/mL), compared with the untreated control (p < 0.05). At 1× MIC, the bacterial count stabilized at approximately 4 log CFU/mL, while in untreated samples, growth increased to approximately 12 log CFU/mL by 24 h. Notably, at concentrations of 2× MIC and higher, no viable cells were detected after 8 h, and this absence of growth persisted throughout the 24 h observation period.

### Cell morphology after treatment with fraction F4

**Figure 5:**
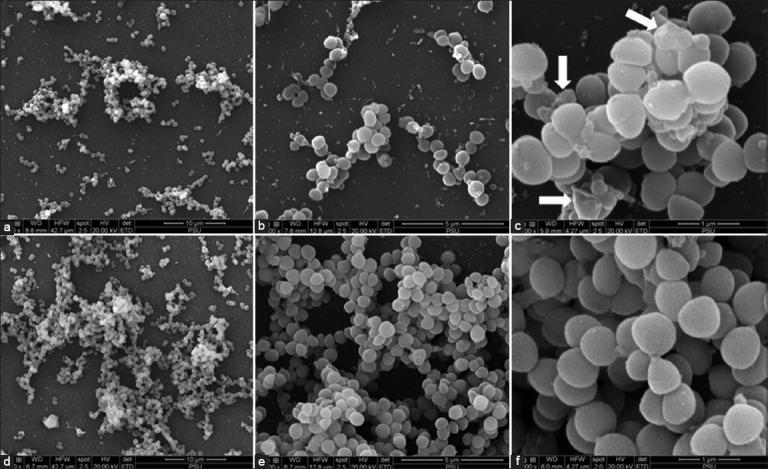
Scanning electron microscopy micrograph of bacterial cells treated with fraction F4 at 2× minimal inhibitory concentration (MIC) (MIC = 0.5 µg/mL) for 3 h (a-c) and untreated *Staphylococcus pseudintermedius* CABI 240105 (d-f). The magnifications are as follows: a, d = 3000×; b, e = 10000×; c, f = 30000×. White arrows indicate abnormal cells.

SEM analysis was performed to assess the morphological effects of F4 on *S. pseudintermedius*. Cells treated with 2× MIC for 4 h exhibited reduced density and pronounced morphological abnormalities compared with untreated controls (Figure 5). Alterations to cell membranes and abnormal cellular structures were observed, indicating damage likely attributable to the action of rhodomyrtone.

### Antibiofilm activity

**Figure 6:**
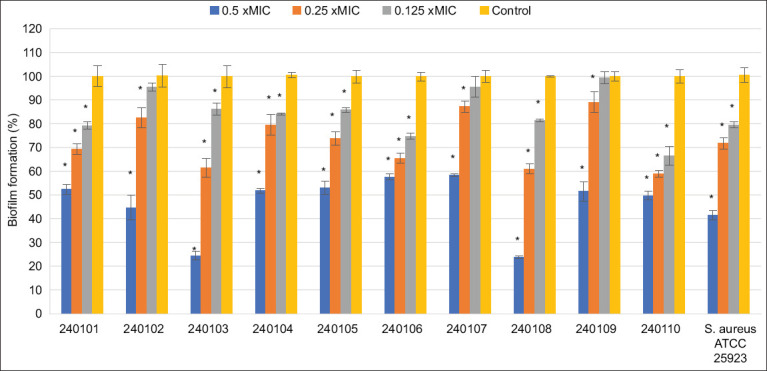
Effects of fraction F4 on biofilm formation of *Staphylococcus pseudintermedius* clinical isolates. *Staphylococcus aureus* ATCC 25923 was used as the referent strain. The biofilms were treated with extract at different concentrations. 1% dimethylsulfoxide was used as a negative control. Data are presented as mean ± standard deviation from at least three independent experiments (p < 0.05). ATCC=American Type Culture Collection.

The antibiofilm potential of fraction F4 was evaluated using the crystal violet assay against ten clinical *S. pseudintermedius* isolates and the reference strain *S. aureus* ATCC 25923 (Figure 6). Sub-inhibitory concentrations (0.5×, 0.25×, and 0.125× MIC) were employed to ensure the compound’s effect was specifically on biofilm inhibition rather than bacterial killing. At all tested concentrations, biofilm formation by *S. aureus* was significantly suppressed (p < 0.05). For *S. pseudintermedius*, biofilm formation was completely inhibited at 0.5× MIC across all isolates. Significant inhibition was also observed in eight isolates at 0.25× MIC. The antibiofilm activity was found to be dose-dependent.

## DISCUSSION

*S. pseudintermedius* is an opportunistic pathogen implicated in wound infections and other complications in companion animals, including soft tissue infections, urinary tract infections, and surgical site infections. Its growing significance as a public health concern stems from increasing resistance to β-lactam antibiotics. To mitigate the rise of antibiotic-resistant strains, this study proposed an alternative therapeutic strategy using natural compounds extracted from *R. tomentosa*. Conventionally, *R. tomentosa* has been employed in folk medicine to treat various ailments, such as diarrhea, endometritis, appendicitis, dysentery, abscesses, and urinary tract infections [10, 28]. The ethanolic leaf extract of *R. tomentosa* has demonstrated potent antibacterial activity, particularly against Gram-positive bacteria. It has also been shown to inhibit the adhesion and invasion of methicillin-resistant *S. aureus* (MRSA) into host cells [13, 29] and has been utilized as a treatment for *S. aureus* infections in cases of bovine mastitis [22].

Rhodomyrtone, a purified compound derived from *R. tomentosa*, has been identified as a key antibacterial agent in the extract. It has been proposed as a promising therapeutic candidate for combating Gram-positive bacterial infections, exhibiting MIC and MBC values against multidrug-resistant *S. aureus* (MRSA) in the range of 0.39–0.78 µg/mL [30]. Notably, its antimicrobial potency is comparable to that of vancomycin, a last-resort antibiotic for drug-resistant staphylococcal infections.

A previous study has shown that rhodomyrtone primarily exerts its antibacterial effects by targeting bacterial cell membranes. In MRSA, treatment with rhodomyrtone leads to leakage of adenosine triphosphate (ATP) and potassium ions, indicative of cytoplasmic membrane disruption [31]. Rhodomyrtone also disturbs membrane potential, resulting in the release of ATP, lipids, and cytoplasmic proteins [32]. The compound induces rapid collapse of membrane potential and decreases membrane integrity [33]. Furthermore, rhodomyrtone’s mechanism is distinct from conventional antibiotics; it transiently binds to phospholipid head groups, disrupting lipid packing and inducing membrane fluidization and curvature [34].

Given the low yield of the purified compound, this study employed fractionation of the ethanolic extract to isolate rhodomyrtone-enriched fractions from *R. tomentosa* leaves. The acetone-soluble extract was subjected to quick column chromatography, yielding five rhodomyrtone-containing fractions (F3-F7) in a single step using a hexane: ethyl acetate (92:8 v/v) elution system. TLC analysis of the selected fractions revealed distinct component profiles, and ^1^H NMR spectral data confirmed the presence of rhodomyrtone. Among these, fraction F4 showed rhodomyrtone as a major constituent.

The antibacterial activity of these five fractions was assessed using broth microdilution assays against *S. aureus* and *S. pseudintermedius*. All fractions effectively inhibited bacterial growth, with MIC and MBC values ranging from 0.5 to 16 µg/mL. Their antimicrobial efficacy was comparable to that of pure rhodomyrtone, as reported in previous studies [30]. These results are likely attributable to the high rhodomyrtone content in the fractions, which was quantified by HPLC. Notably, fraction F4 contained rhodomyrtone at a concentration of 489.08 mg/g, a level that may account for its specific antibacterial activity [35]. These findings suggest that *R. tomentosa* fractions can serve as standardized herbal formulations with strong antibacterial properties.

Based on the MIC, MBC, and rhodomyrtone content data, fraction F4 was selected for further evaluation against ten clinical isolates of *S. pseudintermedius*. This fraction effectively inhibited both planktonic growth and biofilm formation. Although it contained approximately 50% (w/w) rhodomyrtone, its antibacterial activity was nearly equivalent to that of the pure compound. Several previous studies have shown that natural fractions can exhibit similar or even superior efficacy compared to isolated compounds. This may be attributed to synergistic interactions between rhodomyrtone and other components present in the fraction, a phenomenon also observed in studies of rhinacanthin-rich extracts from *Rhinacanthus nasutus* and andrographolide-rich fractions from *Andrographis paniculata* [36, 37].

Bactericidal activity was defined as a reduction of ≥3 log CFU/mL. Fraction F4 exhibited rapid and significant bactericidal effects, reducing *S. pseudintermedius* viability by approximately 4 log CFU/mL (99.99%) within 8 h at concentrations of 2×, 4×, and 8× MIC. No bacterial regrowth was detected throughout the 24-h incubation period. These results are consistent with previous reports on pure rhodomyrtone, which demonstrated a 3-log reduction in *S. aureus* counts within 3 h [15]. In another study, treatment of epidemic MRSA with 8× MIC (MIC = 4 µg/mL) for 10 h led to a 2-log reduction, with similar reductions observed using 2× and 4× MIC over 24 h [38].

SEM analysis confirmed the inhibitory effect of fraction F4. After treatment with 2× MIC (0.5 µg/mL) for 3 h, SEM images revealed fewer bacterial cells and notable morphological abnormalities compared to untreated controls. This time point and concentration were selected to allow visualization of damaged cells without complete eradication. Abnormalities observed on the bacterial surface were consistent with the membrane-targeting action of rhodomyrtone. While no dramatic surface differences were noted between treated and untreated cells, signs of cell wall disruption and altered morphology were evident. These effects may be attributed to the relatively low treatment concentration and short exposure duration. A previous study demonstrated that MRSA treated with rhodomyrtone at 16 µg/mL for 1 h exhibited pore formation and bulging structures on the cell surface [39].

## CONCLUSION

This study demonstrated that fractionation of the ethanolic extract of *R. tomentosa* leaves yields rhodomyrtone-rich fractions with potent antibacterial activity against *S. pseudintermedius*, an emerging multidrug-resistant zoonotic pathogen. Among the tested fractions, F4 exhibited the highest rhodomyrtone content (489.08 mg/g) and the most potent antimicrobial properties, with MIC and MBC values ranging from 0.5 to 2 µg/mL and 2 to 8 µg/mL, respectively, against ten clinical isolates. Fraction F4 also significantly reduced bacterial viability by 4 log CFU/mL (99.99%) within 8 h and effectively inhibited biofilm formation at sub-inhibitory concentrations. SEM confirmed morphological alterations in treated cells, consistent with membrane-targeting mechanisms previously attributed to rhodomyrtone.

The strengths of this study lie in its comprehen-sive approach, including chemical characterization of bioactive fractions, comparative antimicrobial assessments, and the integration of both planktonic and biofilm models. Notably, the antibacterial activity of fraction F4 was comparable to that of pure rhodomyrtone and doxycycline, suggesting that semi-purified herbal formulations may serve as cost-effective alternatives to synthetic antibiotics. This finding is particularly relevant in the context of veterinary medicine, where natural antimicrobial agents are increasingly sought as alternatives to combat antimicrobial resistance.

However, certain limitations should be acknowledged. First, while the study focused on Gram-positive bacteria, its spectrum of activity against Gram-negative pathogens remains uninvestigated. Second, the precise synergistic interactions between rhodomyrtone and other phytochemicals within the fractions were not elucidated. In addition, in vivo efficacy, toxicity, and pharmacokinetic data are lacking, which are essential for translational applications.

Future research should aim to identify and quantify other bioactive constituents within the rhodomyrtone-rich fraction and explore their possible synergistic effects. Investigations into formulation stability, delivery systems, and therapeutic efficacy in animal models of infection are warranted. Expanding the antimicrobial screening to include Gram-negative bacteria and resistant biofilm-associated infections would also enhance the clinical relevance of this work. Collectively, these findings support the development of rhodomyrtone-rich fractions as standardized, plant-based antimicrobial agents for managing drug-resistant staphylococcal infections in veterinary settings.

## AUTHORS’ CONTRIBUTIONS

MD and SP: Conceived and designed the study and evaluated the laboratory work. MD, SP, and SL: Analyzed and interpreted the data and drafted the manuscript. SC: Conceived and designed the study. SPV: Supervised the study and reviewed and edited the manuscript. All authors have read and approved the final manuscript.
